# Cordycepin Inhibits Lipopolysaccharide (LPS)-Induced Tumor Necrosis Factor (TNF)-α Production via Activating AMP-Activated Protein Kinase (AMPK) Signaling

**DOI:** 10.3390/ijms150712119

**Published:** 2014-07-08

**Authors:** Jian-Li Zhang, Ying Xu, Jie Shen

**Affiliations:** 1 Department of Respiration, the First Affiliated Hospital of Zhejiang University School of Medicine, Hangzhou 310003, China; E-Mail: jianlizhangjj@163.com; 2 Department of Pediatrics, the First People’s Hospital of Hangzhou, Hangzhou 310003, China; E-Mail: yingyingxuxu163@163.com; 3 Department of Pediatrics, the First Affiliated Hospital of Zhejiang University School of Medicine, Hangzhou 310003, China

**Keywords:** Kawasaki disease, cordycepin, TNFα (tumor necrosis factor α), AMPK (AMP-activated protein kinase), LPS (lipopolysaccharide)

## Abstract

Tumor necrosis factor (TNF)-α is elevated during the acute phase of Kawasaki disease (KD), which damages vascular endothelial cells to cause systemic vasculitis. In the current study, we investigated the potential role of cordycepin on TNFα expression in both lipopolysaccharide (LPS)-stimulated macrophages and *ex vivo* cultured peripheral blood mononuclear cells (PBMCs) of KD patients. We found that cordycepin significantly suppressed LPS-induced TNFα expression and production in mouse macrophages (RAW 264.7 cells and bone marrow-derived macrophages (BMDMs)). Meanwhile, cordycepin alleviated TNFα production in KD patients’ PBMCs. PBMCs from healthy controls had a much lower level of basal TNF-α content than that of KD patients. LPS-induced TNF-α production in healthy controls’ PBMCs was also inhibited by cordycepin. For the mechanism study, we discovered that cordycepin activated AMP-activated protein kinase (AMPK) signaling in both KD patients’ PBMCs and LPS-stimulated macrophages, which mediated cordycepin-induced inhibition against TNFα production. AMPK inhibition by its inhibitor (compound C) or by siRNA depletion alleviated cordycepin’s effect on TNFα production. Further, we found that cordycepin inhibited reactive oxygen species (ROS) production and nuclear factor kappa B (NF-κB) activation in LPS-stimulate RAW 264.7 cells or healthy controls’ PBMCs. PBMCs of KD patients showed higher basal level of ROS and NF-κB activation, which was also inhibited by cordycepin co-treatment. In conclusion, our data showed that cordycepin inhibited TNFα production, which was associated with AMPK activation as well as ROS and NF-κB inhibition. The results of this study should have significant translational relevance in managing this devastating disease.

## 1. Introduction

Kawasaki disease (KD), the acute febrile disease in children, is characterized by systemic vasculitis, which will lead to coronary artery lesions and other serious cardiovascular complications [1,2,3]. The abnormal activation of immunocompetent cells (*i.e.*, monocytes, macrophages and lymphocytes) is the main feature of KD. These inflammatory cells could synthesize and secrete various pro-inflammatory cytokines and chemokines, *i.e.*, tumor necrosis factor (TNF)-α, interferon (IFN)-γ and interleukin 6 (IL-6), to activate endothelial cells causing vasculitis [4,5,6,7]. 

Despite appropriate therapies, coronary artery aneurysms continue to develop in many affected KD patients, it is also the leading cause of acquired heart disease in children. The role of TNFα in the vascular inflammation of KD is well-established: TNFα content is significantly increased in the peripheral blood of KD patients during the acute phase [4,5,6,7]. TNFα is a potent inflammatory cytokine that causes damage to vascular endothelial cells, it is becoming an important contributor of the pathogenesis of both the immune activation and endothelial cell damage in KD patients [4,5,6,7]. 

TNF-α could activate endothelial cells through the increased expression of adhesion molecules including intercellular adhesion molecule-1 (ICAM-1), vascular cell adhesion molecule-1 (VCAM-1), and E-selectin [8,9,10]. TNF-α is also shown to up-regulate the expression of chemokines such as macrophage inflammatory protein 1α (MIP-1α) and RANTES; these chemokines are important in the orchestration of leukocyte-endothelial interactions leading to vascular endothelium activation [8,9,11,12]. In an animal model of KD, it has been shown TNFα is required for the development of coronary artery lesions [13]. Mice treated with the TNF-α-blocking agent (etanercept), or depleted with the TNF-α receptor, were resistant to development of both coronary arteritis and coronary aneurysm formation in the KD mice model [13].

TNFα blocking agents have been investigated in isolated cases of KD patients [14]. Burns *et al.* [15] showed KD patients responded rapidly and completely to a single infusion of the anti-TNF-α monoclonal antibody, infliximab. Oishi *et al.* [16] administered infliximab to a one-month-old girl with refractory KD and coronary artery aneurysm; the authors found that the coronary artery aneurysm improved and KD was controlled without complications 20 months after the onset. These results suggest a critical role of TNF-α in KD pathogenesis. Our previous study has demonstrated that perifosine, a novel Akt inhibitor, significantly inhibited TNFα production via activating of AMP-activated protein kinase (AMPK) signaling and inhibiting Erk activation [17].

Cordycepin (3'-deoxyadenosine) is a bioactive compound present in species of fungi belonging to the genus Cordyceps [18,19,20,21]. Cordycepin is reputed to exert a large variety of biological functions, including cell proliferation inhibition, apoptosis induction, platelet aggregation inhibition, cell migration and invasiveness interference and inflammation suppression [18,19,20,21,22,23,24]. In the current study, we explored the role of cordycepin on TNFα production in both lipopolysaccharide (LPS)-stimulated macrophages and KD patients’ peripheral blood mononuclear cells (PBMCs).

## 2. Results and Discussion

### 2.1. Sub-Cytotoxic Cordycepin Inhibits LPS (Lipopolysaccharide)-Induced TNFα (Tumor Necrosis Factor α) Production in RAW 264.7 Mouse Macrophages

**Figure 1:**
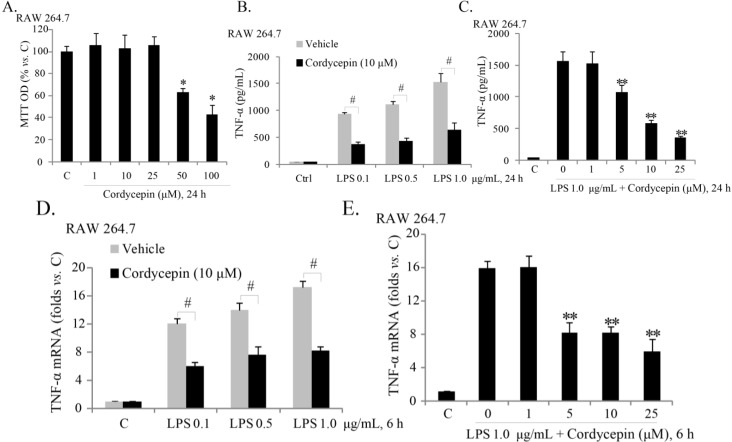
Sub-cytotoxic cordycepin inhibits LPS (lipopolysaccharide)-induced TNFα (tumor necrosis factor α) production in RAW 264.7 mouse macrophages. RAW 264.7 mouse macrophages were either left untreated (“C”), or treated with the indicated concentration of cordycepin (0–100 μM) for 24 h, cell viability was analyzed by MTT (Thiazolyl Blue Tetrazolium Bromide) assay (**A**); RAW 264.7 cells were stimulated with the indicated concentration of LPS, co-supplemented with the indicated cordycepin for 24 h; TNFα in culture supernatant was measured with an ELISA kit (R&D Systems, Shanghai, China) (**B**,**C**); TNFα mRNA expression in RAW 264.7 cells with the indicated treatment was analyzed by real-time PCR (**D**,**E**). The results presented were representative of three independent experiments. The values were expressed as the means ± SD. * *p* < 0.05 compared with “C” group (**A**); ** *p* < 0.05 compared with LPS only group (**C**,**E**); ^#^ *p* < 0.05 (**B**,**D**).

Since cordycepin has been investigated as an anti-cancer drug [25,26], we here explored the potential role of cordycepin on LPS-induced TNFα production in macrophages. Thus, we first examined whether cordycepin affects the cell survival of RAW 264.7 mouse macrophages. Cordycepin did not show a significant cytotoxic effect in RAW 264.7 cells except at high doses (50–100 μM) (Figure 1A). 

TNFα ELISA results in Figure 1B demonstrated that cordycepin (10 μM) significantly inhibited LPS (0.1–1.0 μg/mL)-induced TNFα production in RAW cells, and the effect of cordycepin on TNFα production was dose-dependent (Figure 1C). Further, as shown in supplementary Figure 1A, cordycepin (10 μM) inhibited LPS (0.1 μg/mL)-induced TNFα production over a 72-h period, and the 24-h time point was the optimal duration of treatment. No cell viability decrease was noted over the 72-h cordycepin (10 μM) + LPS (0.1 μg/mL) treatment in RAW cells (Figure S1B). Meanwhile, as shown in Figure 1D, cordycepin dramatically suppressed LPS-induced TNFα mRNA up-regulation in RAW cells, and the effect of cordycepin was again dose-dependent (Figure 1E). These results show that sub-cytotoxic cordycepin attenuates LPS-induced TNFα mRNA expression and production in RAW 264.7 mouse macrophages.

### 2.2. Cordycepin Inhibits TNFα Production in ex-Vivo Cultured Peripheral Blood Mononuclear Cells (PBMCs) of KD (Kawasaki Disease) Patients

**Figure 2:**
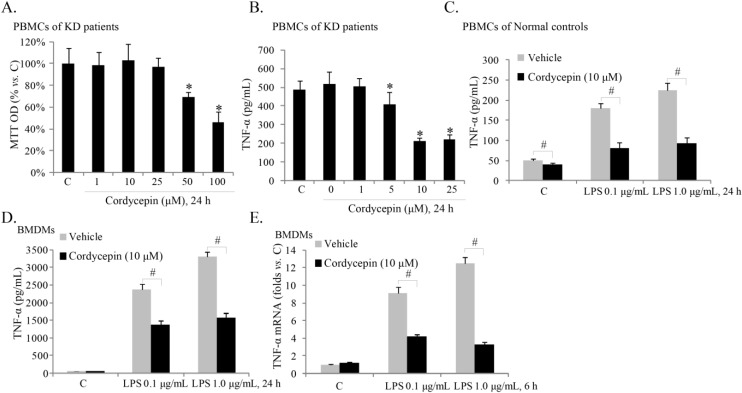
Cordycepin inhibits TNFα production in *ex vivo* cultured peripheral blood mononuclear cells (PBMCs) of KD (Kawasaki disease) patients. The *ex vivo* cultured PBMCs of acute KD patients were either left untreated (“C”) or treated with indicated concentrations of cordycepin for 24 h; cell viability was analyzed by MTT assay (**A**); TNFα in culture supernatant was measured with the ELISA kit (**B**); The *ex vivo* cultured PBMCs from healthy controls or the primary BMDMs (bone marrow derived macrophages) were stimulated with LPS (0.1 or 1.0 μg/mL) in the presence or absence of cordycepin (10 μM). TNFα content in culture supernatant was measured with the ELISA kit 24 h after treatment (**C**,**D**); and TNFα mRNA expression was examined 6 h after stimulation (**E**, for BMDMs). The results presented are representative of three independent experiments. The values are expressed as the means ± SD. * *p* < 0.05 compared with “C” group (**A**,**B**); ^#^ *p* < 0.05 (**C**–**E**).

Increased TNFα production from PBMCs of acute KD patients is the main cause of vasculitis. Next, we tested if cordycepin affected TNFα levels in PBMCs of KD patients. Again, viability of PBMC cells was not affected by low-doses of cordycepin treatment (<50 μM) (Figure 2A). Similarly to what we have previously reported [17], the ELISA results in Figure 2B demonstrated a high basal TNFα content in the medium of *ex vivo* cultured PBMCs of acute KD patients. Significantly, sub-cytotoxic cordycepin dose-dependently decreased TNFα content in the medium of the PBMCs (Figure 2B). PBMCs from healthy controls showed a much lower level of basal TNF-α content than that of KD patients (Figure 2C). Further, LPS-induced TNF-α production in the PBMCs froom healthy controls was also inhibited by cordycepin (Figure 2C). We also tested the effect of cordycepin on LPS-induced TNFα expression and production in primary cultured mouse bone marrow derived macrophages (BMDMs), and results clearly demonstrated that cordycepin inhibited LPS-induced TNFα mRNA expression (Figure 2D) and protein secretion (Figure 2E) in BMDMs. 

### 2.3. AMPK (AMP-Activated Protein Kinase) Activation Is Required for Cordycepin’s Effect of LPS-Induced TNFα Production

**Figure 3:**
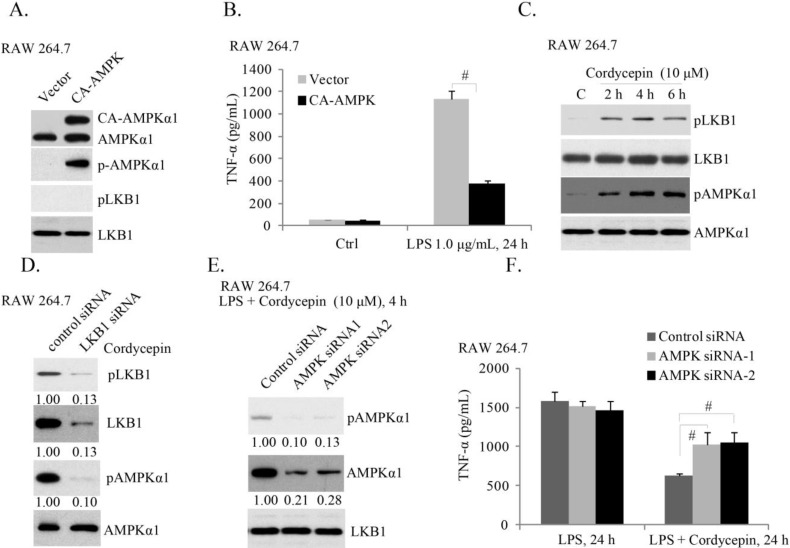
AMPK (AMP-activated protein kinase) activation is required for cordycepin’s effect of LPS-induced TNFα production. The stable RAW 264.7 cells expressing empty vector (GFP) or constitutively-active (CA)-AMPKα-GFP (T172D) were stimulated with LPS (1.0 μg/mL) for 24 h, expression of p-AMPKα, AMPKα, p-LKB1 and LKB1 was tested by Western blots (**A**); TNFα in culture supernatant was also measured (**B**); RAW 264.7 cells were treated with cordycepin (10 μM) for the indicated time; p-AMPKα, AMPKα, p-LKB1 and LKB1 were tested (**C**); RAW 264.7 cells transfected with scramble siRNA (100 nM, 48 h) or LKB1 siRNA (100 nM, 48 h) were treated with cordycepin (10 μM) for 4 h, p-AMPKα; AMPKα, p-LKB1 and LKB1 were tested (**D**); RAW 264.7 cells transfected with scramble siRNA (100 nM, 48 h) or AMPKα siRNAs (−1 and −2) (100 nM, 48 h) were treated with LPS (1.0 μg/mL), or plus cordycepin (10 μM, LPS + cordycepin); expression of p-AMPKα, AMPKα and LKB1 was tested 4 h after LPS stimulation (**E**); and TNFα in culture supernatant was also measured 24 h after LPS stimulation (**F**). Results presented are representative of three independent experiments; the values are expressed as the means ± SD. ^#^ *p* < 0.05 (**B**,**F**).

Activation of AMPK is known to exert an anti-inflammatory effect [27,28]. Our previous study showed that AMPK activation by its activators AICAR and A769662, or by perifosine, significantly inhibited LPS-induced TNFα production in mouse macrophages and KD patients’ PBMCs [17]. To exclude the off-target effect of these reagents, we introduced the constitutively-active (T172D) AMPKα (CA-AMPKα) [29] into RAW cells, and created a stable cell line (Figure 3A). ELISA results in Figure 3B showed that CA-AMPK suppressed LPS-induced TNFα production, further supporting the inhibitory role of AMPK activation on TNFα production [17]. It has been shown that cordycepin activates AMPK mainly in cancer cells [30]. Thus, we tested its effect in macrophages. As shown in Figure 3C, cordycepin induced significant AMPK and its upstream LKB1 phosphorylation in RAW cells, while LKB1 reduction by siRNA (causing more than 87% reduction of LKB1 expression) inhibited cordycepin-induced AMPK phosphorylation, indicating that LKB1 might be the upstream kinase for AMPK activation by cordycepin (Figure 3D). Importantly, AMPK siRNA knockdown dramatically attenuated cordycepin’s effect on TNFα production in RAW cells (Figure 3F). Note that we utilized two non-overlapping siRNAs against different mRNA sequence of AMPK (siRNA-1, siRNA-2), and observed similar results (Figure 3E). These results suggest that activation of AMPK is required for cordycepin-induced inhibition of TNFα production induced by LPS.

### 2.4. Cordycepin Activates AMPK to Inhibit TNFα Production in PBMCs of KD Patients

**Figure 4:**
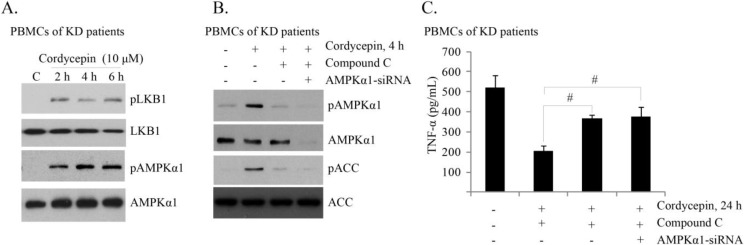
Cordycepin activates AMPK to inhibit TNFα production in PBMCs of KD patients. The *ex vivo* cultured PBMCs of acute KD patients were either left untreated (“C”), or treated with cordycepin (10 μM) for indicated time; expression of p-AMPKα, AMPKα, p-LKB1, LKB1 was tested by Western blots (**A**); The effect of compound C (10 μM, 1 h pretreatment), and AMPK siRNA-1 (100 nM, 48 h) on cordycepin (10 μM)-induced AMPK/ACC phosphorylation (4 h) and TNFα production (24 h) in KD patients’ PBMCs were tested by western blots (**B**) and ELISA (**C**), respectively. The results presented are representative of three independent experiments. The values are expressed as the means ± SD. ^#^ *p* < 0.05 (**C**).

Next, we tested the effect of cordycepin in PBMCs of KD patients. As shown in Figure 4A,B, cordycepin activated AMPK signaling in PBMCs of KD patients, as p-AMPKα/p-LKB1/p-ACC (acetyl-CoA carboxylase) was induced in cordycepin-stimulated cells, which was inhibited by its inhibitor compound C or AMPKα siRNA (Figure 4B) in PBMCs of KD patients. Significantly compound C and AMPKα siRNA alleviated cordycepin’s inhibitory effect on TNFα production in PBMCs of KD patients (Figure 4C). Together, these results indicate that AMPK activation is important for cordycepin-mediated anti-TNFα production effect in PBMCs of KD patients. 

### 2.5. Cordycepin Inhibits ROS (Reactive Oxygen Species) Production and NF-κB (Nuclear Factor Kappa B) Activation in Both LPS-Stimulated RAW 264.7 Cells and PBMCs from KD Patients

**Figure 5:**
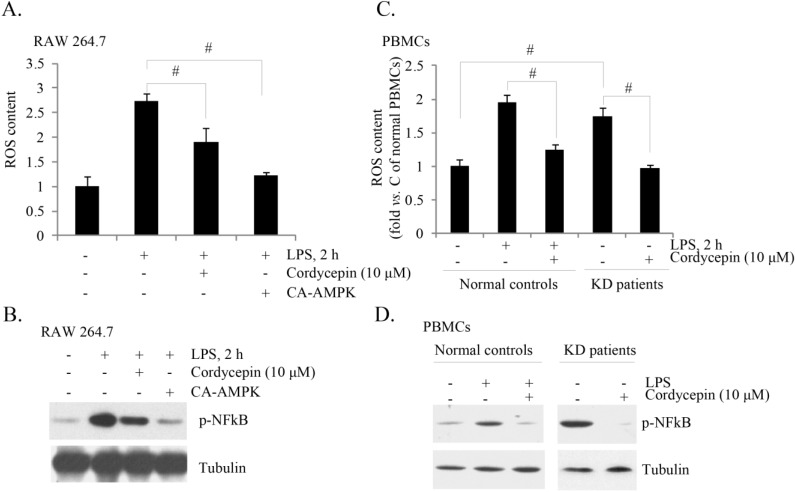
Cordycepin inhibits ROS (reactive oxygen species) production and NF-κB activation in both LPS-stimulated RAW 264.7 cells and KD patients’ PBMCs. RAW 264.7 cells were pretreated with cordycepin (10 μM, 1 h) or CA-AMPK (2 μg/mL, 48 h), followed by LPS (1.0 μg/mL) stimulation for 2 h, ROS production and p-NFκB/tubulin expression were tested by FACS (**A**) and western blotting (**B**), respectively. The *ex vivo* cultured PBMCs of KD patients/healthy controls were treated with cordycepin (10 μM), with or without LPS (1.0 μg/mL) for 2 h; ROS production and p-NFκB/tubulin expression were tested (**C**,**D**). The results presented were representative of three independent experiments. The values were expressed as the means ± SD. ^#^ *p* < 0.05 (**A**,**B**).

LPS is known to induce ROS production, which plays an important role in subsequent NF-κB activation and TNF-α production [31,32,33]. A recent study by Jeon *et al.* [34] has established the anti-oxidant function of AMPK activation. Activated AMPK phosphorylates and inhibits its downstream target ACC, thus decreasing nicotinamide adenine dinucleotide phosphate (NADPH) consumption in fatty-acid synthesis and increasing NADPH generation by means of fatty-acid oxidation [34]. Thus, AMPK activation could serve as an important mechanism of anti-oxidant. We have shown that cordycepin activated AMPK and inhibited LPS-induced TNF-α production. Here, we found that LPS-induced ROS production and NF-κB activation were also inhibited by cordycepin, and by CA-AMPKα (Figure 5A,B) in RAW cells. In *ex vivo* cultured PBMCs of KD patients, we observed high base levels of ROS production and NF-κB activation (as compared to PBMCs of healthy controls), which were both inhibited by cordycepin (Figure 5C,D). Meanwhile, LPS-induced ROS production and p-NFkB induction in PBMCs from healthy controls were also inhibited by cordycepin (Figure 5C,D). Note that NF-κB activation was reflected by p-NFκB (Figure 5B,D). Based on these results, we propose that cordycepin-induced inhibition of TNF-α might be associated with its functions on ROS scavenging and NF-κB in-activation.

## 3. Material and Methods

### 3.1. Chemical and Regents

Cordycepin, LPS and compound C were purchased from Sigma (Shanghai, China). LPS was dissolved in sterile, pyrogen-free water with repeated vortexing to yield a stock solution of 1 mg/mL and this was stored at −20 °C. Cordycepin was dissolved in sterile PBS (phosphate-buffered saline) at 5 mM. Both agents were diluted to the indicated concentrations of culture medium.

### 3.2. Antibodies

Anti-AMPKα1, tubulin, acetyl-CoA carboxylase (ACC), LKB1, rabbit and mouse horseradish peroxidase (HRP)-conjugated IgG antibodies were purchased from Santa Cruz (Santa Cruz, CA, USA). Antibodies against phospho (p)-AMPKα (Thr 172), p-ACC (Ser 79), p-nuclear factor kappa B (NF-κB, Ser 536) and p-LKB1 (Ser 428) were purchased form Cell Signaling Tech (Denver, MA, USA). The dilution of the primary antibodies used in this study was 1:500–1:1000. 

### 3.3. RAW 264.7 Mouse Macrophage Culture

As previously reported [17], RAW 264.7 cells (ATCC, Rockville, MD, USA) were cultured in Dulbecco’s modified Eagle medium (DMEM) supplemented with 10% fetal bovine serum (FBS), 100 U/mL streptomycin, and 2 mM glutamine at 37 °C in a 5% CO_2_ humidified incubator. 

### 3.4. Bone Marrow–Derived Macrophages (BMDMs) Culture

As previously reported [17], the bone marrow was flushed from femurs of mice (2 month old) with 7 mL of DMEM supplemented with 10% FBS. Cell pellets were resuspended in ACK hypotonic buffer (Hao-ran Biotech, Shanghai, China) to remove red blood cells, and were subsequently washed with DMEM with 10% FBS and cultured at the concentration of 10^7^ cells/mL in DMEM supplemented with 20% FBS and 30% L929 cell conditioned media. Six to seven days later, adherent macrophages were trypsinized, counted, and re-plated for experimental use. Prior to stimulation, BMDMs were cultured overnight in DMEM supplemented with only 0.5% FBS.

### 3.5. Ex Vivo Culture of Peripheral Blood Mononuclear Cells (PBMCs)

As previously reported [17], PBMCs of three acute KD patients (each patient at the acute phase, and each patient’s PBMCs were used for one set of experiments) and three comparable healthy controls (same age, same sex) were isolated by centrifugation over lymphocyte separation medium (Biotyme, Shanghai, China). After three washes in PBS, the PBMCs were counted and cultured in DMEM supplemented with 10% FBS, 2 μg of phytohemagglutinin (PHA) per mL, 10 ng of phorbol 12-myristate-13-acetate per mL, nonessential amino acids, 5 mM β-mercaptoethanol, 10 mM HEPES, 2 mM glutamine, 1 mM sodium pyruvate, 100 U of penicillin per mL, and 100 μg of streptomycin per mL. The study was approved by the institutional review board of the authors’ institutions, and written informed consent was obtained from each acute KD patient. All patients’ investigations were conducted according to the principles expressed in the Declaration of Helsinki.

### 3.6. Cell Viability Assay

Cell viability was measured by the 3-[4,5-dimethylthylthiazol-2-yl]-2,5diphenyltetrazolium bromide (“MTT”) assay as reported [17]. Briefly, cells were cultured and seeded in 96-well plates. After treatments, twenty μL/well of MTT working solution (5 mg/mL) was added to each well, and were incubated continuously at 37 °C for 3 h. The culture supernatant was removed from the wells very carefully, and DMSO (150 μL/well) was added to dissolve the formazan crystals. The absorbance of each well was measured at 490 nm with an ELISA reader (Molecular Devices, Sunnyvale, CA, USA). The value of treated group was normalized to that of the control group.

### 3.7. TNFα ELISA (Enzyme-Linked Immunosorbent Assay) Assay

After treatment, TNFα protein content in culture supernatant was measured with a TNFα ELISA kit (R&D Systems), according to the manufacturer’s instructions. The concentrations of TNFα in each sample were calculated from a standard curve prepared using known concentrations of recombinant TNFα (R&D Systems).

### 3.8. Western-Blots

Cells with the indicated treatment were harvested in the lysis buffer (Jing-mei Biotech, Shanghai, China). The protein concentration was determined by Bio-Rad protein assay (Bio-Rad, Beijing, China). Aliquots of 30 µg of lysates were electrophoresed on 10% SDS-PAGE gel and transferred to a PVDF (polyvinylidene fluoride) membrane. The blot was incubated in the blocking buffer (10% milk in PBS + 0.05% Tween 20 (PBST)), and then incubated with the primary antibody at 4 °C overnight with PBST. Appropriate secondary antibody conjugated to horseradish peroxidase (HRP) was then added. Antigen-antibody complex was detected by using enhanced chemiluminescence (ECL) reagent. All Western-blots in this study were subjected to different exposures: from 10 s to 30 min, the best exposures were selected for data presentation. When indicated, the blot intensity was quantified through the Image J software (National Institute of Healthy, Bethesda, MD, USA) before normalization to the corresponding loading control.

### 3.9. Total RNA Isolation and Real-Time Reverse Transcriptase Polymerase Chain Reaction (RT-PCR)

After treatment, cells were washed with 1 mL of PBS, and RNA was extracted using the RNA-TRIZOL extraction reagent (Gibco, Shanghai, China), according to the manufacturer’s instructions. Extracted RNA was quantified using a NanoDrop ND1000 spectrophotometer (Labtech International, Ringmer, UK), and the purity of each sample was determined by the ratio A_260_/A_280_. An A_260_/A_280_ ratio of between 1.8 and 2.1 was considered suitable for further investigation. For cDNA synthesis, RNA samples were treated with DNase I and reverse-transcribed into cDNA using oligo-dT primers (Oligo, Warsaw, Poland). Reverse transcription was performed through the TOYOBO ReverTra Ace RT-PCR kit (TAKARA BIO Inc., Tokyo, Japan) using SYBR Green I as detection dye, according to the manufacturer’s protocols. To create a standard curve, six 10-fold serial dilutions of the 1 × 10^6^ of cDNA were used. The cycle threshold was recorded and plotted as a function of the dilution to generate a straight line with a slope that was related to the doubling efficiency. The efficiency raised to the value of the intercept of the line at no dilution is a measure of the relative copy number of cDNA for each gene in samples. The primers (forward: 5'-ATGAGCACTGAAAGCATGATC-3'; reverse: 5'-CAGATGACCTAGTAACGGACT-3') were for TNFα. The primers (forward: 5'-CAATGACCCCTTCATTGACC-3'; reverse: 5'-GACAAGCTTCCCGTTCTCAG-3') were for glyceraldehyde-3phosphate dehydrogenase (GAPDH). A typical reaction (50 μL) contained 1/50 of reverse transcription–generated cDNA and 200 nM of primer in 1× SYBR Green RealTime Master Mix buffer (Toyobo, Shanghai, China). The PCR reactions were carried out on a Bio-Rad IQ5 multicolor detection system by using 2 μg of synthesized cDNA under the following conditions: 95 °C for 5 min, 40 cycles at 95 °C for 15 s, 60 °C for 15 s, and 72 °C for 30 s. One RNA sample of each preparation was processed without RT-reaction to provide a negative control in subsequent PCR. After amplification, melt curve analysis was performed to analyze product melting temperature. The GAPDH gene was chosen as the reference gene for normalization, and the 2^−ΔΔ*C*t^ method [35] was applied to quantify TNFα mRNA change within samples. The fold change of TNFα mRNA expression = 2^−ΔΔ*C*t^; Where ΔΔ*C*_t_ = (*C*_t_ TNFα − *C*_t_ GAPDH) of treatment group − (*C*_t_ TNFα − *C*_t_ GAPDH) of control group. (*n* = 3). 

### 3.10. siRNA

The RNAi sequences (5'-GCAUAUGCUGCAGGUAGAU-3' [21] and 5'-AAGGAAAGTGAAGGTGGGCAA-3' [22]) against human AMPK-α1/2 were synthesized by GENEWIZ, Inc. (Suzhou, China). The LKB1 siRNA was purchased from Santa Cruz. Non-sense control RNAi was also purchased from Santa Cruz, and was used as RNAi-negative control. Transient transfections were performed on 6-well plates using FuGENE6 reagent (Roche Molecular Biochemicals, Shanghai, China) according to the manufacturer’s instructions, see [17].

### 3.11. Constitutively Active-AMPK (CA-AMPK) Transfection and Stable Cell Line Selection

The adenoviral vector expressing a constitutively active mutant of AMPKα1 (T172D, Ad-CA-AMPKα1-GFP-puromycin) and the empty vector expressing green fluorescence protein (Ad-GFP) were gifts from Zheng *et al.* [29]. Preliminary studies revealed that within 48 h of transfection with control Ad-GFP, 35%–45% of RAW cells expressed GFP. CA-AMPKα1 or the control vector (2 μg/mL) was transfected by Lipofectamine 2000 (Invitrogen, Carlsbad, CA, USA) according to the manufacturer’s protocol. The stable cell lines were selected by puromycin (1 μg/mL) for 10 days. After selection, more than 95% of cells were GFP positive. Western blots were also utilized to test the transfection efficiency in stable cells.

### 3.12. Reactive Oxygen Species (ROS) Assay

Intracellular ROS generation was measured by flow cytometry using dichlorofluorescin (DCF) oxidation assay. DCFH-DA enters passively into cells and is cleaved by nonspecific cellular esterases and oxidized in the presence of ROS. The cells were first seeded onto 6-cm culture plates and allowed to grow overnight to reach an approximate confluence of 80%. After treatment, the cells were first seeded onto 6-cm culture plates and allowed to grow overnight to reach an approximate confluence of 80%. After treatment, the cells were further incubated with 25 μM DCFH-DA at 37 °C in a CO_2_ incubator for 30 min. The cultured cells were washed, trypsinized and resuspended in PBS. The fluorescence intensity was quantified using a FACSVantage flow cytometric analyzer (BD Bioscience, Heidelberg, Germany), with an excitation wavelength of 488 nm and emission wavelength of 525 nm. For each assay, 10,000 events were analyzed. The fluorescent value of treatment group was normalized as fold changes of the control group, and was presented as the mean ± SD of at least three experiments.

### 3.13. Data Analysis

Data were collected using a minimum of three experiments and used to calculate the mean ± SD. Statistical differences were analyzed by one-way analysis of variance (ANOVA) followed by multiple comparisons performed with post hoc Bonferroni test (SPSS version 18, John Wiley & Sons, Inc., New York, NY, USA). Values of *p* < 0.05 were considered statistically significant. The significance of any differences between two groups was tested using paired-samples *t* test.

## 4. Conclusions

In the current study, we found that cordycepin inhibited LPS-induced TNFα expression and production in mouse macrophages (RAW cells and primary BMDMs). Meanwhile, TNFα content from *ex vivo* cultured PBMCs of acute KD patients was also suppressed by cordycepin. Cordycepin activated AMPK signaling in both patients’ PBMCs and LPS-stimulated macrophages, while inhibition of AMPK (by compound C or siRNA) alleviated cordycepin’s effect on TNFα production. We observed that cordycepin inhibited ROS production and NF-κB activation in both LPS-stimulated RAW 264.7 cells and PBMCs of KD patients, which might be attributed to its inhibitory role on TNFα production.

TNF-α expression is increased in the peripheral blood of KD patients during the acute phase to cause coronary artery lesions [4,6,7]. TNFα blocking agents have been used as salvage therapy in isolated cases of KD patients, and have shown promising results [14,16,36]. In a murine model of KD, TNF-α was rapidly produced by the PBMCs localized to affected coronary vessel walls [13]. The TNF-α production in affected coronary arteries was correlated with the development of the local inflammatory response in the vessel wall [13]. Increased production of TNF-α by PBMCs in the coronary arteries was shown to cause lymphocytes recruitment, leading to a sustained local immune responses coupled with elastin degradation, vessel wall damage, and the characteristic coronary artery lesions seen in KD [13]. On the other hand, blocking TNF-α activity by administration of etanercept, or by TNF receptor depletion, resulted in complete resistance to both inflammation and elastin breakdown in the coronary arteries [13]. Thus, TNF-α activity is necessary for both local inflammation and tissue damage of the coronary arteries in the murine model of KD. The results of this study, that cordycepin significantly decreased TNFα content of *ex vivo* cultured PBMCs of acute KD patients, suggest that cordycepin might be investigated as a possible anti-TNFα agent for KD therapy. 

AMPK acts as a sensor of cellular energy status capable of regulating vital metabolic pathways in the cell [37]. AMPK is activated by conditions that increase the AMP/ATP ratio [37]. Recent studies including ours [17] have explored the potential role of AMPK in modulating inflammatory responses, and have established AMPK as an anti-inflammatory molecule [17,27,38,39]. AICAR and A769662, the AMPK activators, are shown to suppress LPS-mediated pro-inflammatory cytokines production by inhibiting NF-κB nuclear translocation [17,27]. Meanwhile, metformin attenuates the cytokine-induced expression of pro-inflammatory and adhesion molecule genes by activation of AMPK and inhibiting NF-κB activation [28]. Our recent study showed that AMPK activation is involved in perifosine’s inhibitory effect on TNF-α production. In the current study, we found that AMPK was activated by cordycepin in both patients’ PBMCs and LPS-stimulated macrophages, which was important for its role on TNF-α production. The results of this study provide further evidence to support the theory of AMPK signaling regulation for possible KD control [17,27].

Recent studies have shed lights on how AMPK activation attenuates oxidative stress. Activated AMPK could promote NADPH synthesis while limiting its consumption [34]. Thus, in addition to its function in ATP homeostasis, the AMPK/ACC signaling axis works as an anti-oxidant through maintaining NADPH level, and to decrease ROS accumulation [34]. Since cordycepin induced AMPK activation significantly, it was not surprising to see that ROS production was inhibited by cordycepin in both KD patients’ PBMCs and LPS-stimulated macrophages. Further, ROS plays an important role in LPS-induced NF-κB activation and pro-inflammatory factor production [40,41], which might explain why we observed cordycepin’s inhibition on NF-κB activation, and TNF-α production. Thus, we propose that cordycepin induces AMPK activation, which reduces ROS production and suppresses subsequent NF-κB activation, eventually inhibiting TNF-α production. 

We conclude that cordycepin significantly inhibits TNFα production in monocytes, probably via regulating AMPK signaling pathways. The results of this study should have significant translational relevance in managing KD. 

## Supplementary Files

Supplementary File 1
