# Supplementary material for: Cordycepin Inhibits Lipopolysaccharide (LPS)-Induced Tumor Necrosis Factor (TNF)-α Production via Activating AMP-Activated Protein Kinase (AMPK) Signaling
Source: Int J Mol Sci. 2014 Jul 8;15(7):12119–34. doi: 10.3390/ijms150712119 (PMC4139833; doi:10.3390/ijms150712119)

Supplementary Information

Figure S1. RAW 264.7 cells were either left untreated, or stimulated with 0.1 μg/mL of LPS (lipopolysaccharide), co-supplemented with cordycepin (10 μM) for indicated time points; TNFα (tumor necrosis factor α) in culture supernatant was measured with an ELISA kit (**A**); and cell viability was analyzed by MTT (Thiazolyl Blue Tetrazolium Bromide) assay (**B**). The results presented were representative of three independent experiments. The values were expressed as the means ± SD. $^{\#}$ $p < 0.05$.

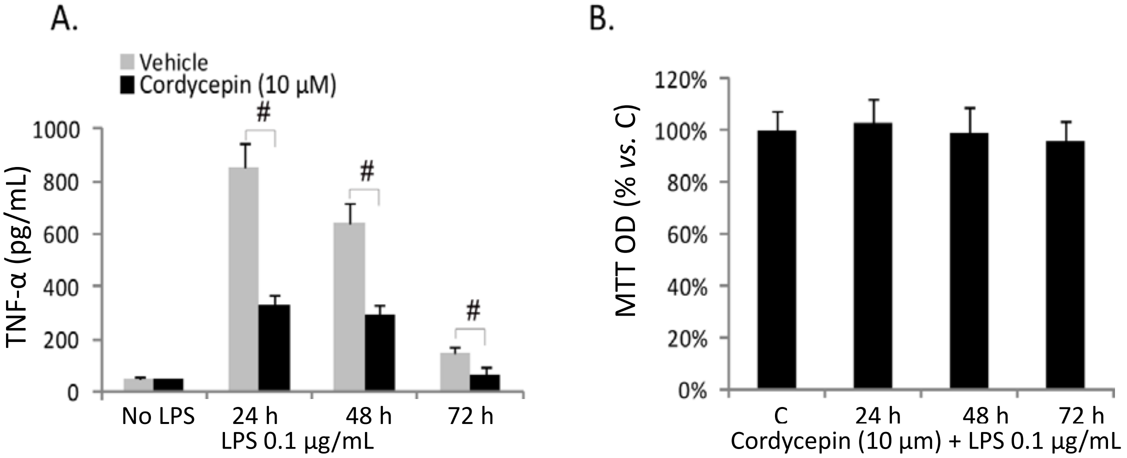

Supplement: Supplementary File 1 [file ijms-15-12119-s001.pdf]
